# A bullous variant of central serous chorioretinopathy treated with oral spironolactone

**DOI:** 10.5935/0004-2749.20230033

**Published:** 2023

**Authors:** David Leonardo Cruvinel Isaac, Leonardo Lando, Hugo Mendes Silva, Marcos P. Avila

**Affiliations:** 1 Retina Service, Department of Ophthalmology, Universidade Federal de Goiás, Goiânia, Brazil

**Keywords:** Retinal detachment, Central serous chorioretinopathy, Retinal pigment epithelium detachment, Spironolactone, Aldosterone antagonist, Descolamento da retina, Coriorretinopatia serosa central, Descolamento do epitélio pigmentado da retina, Espironolactona, Antagonista da aldosterona

## Abstract

We report the case of a 39-year-old male patient who presented with visual loss in the right eye for 6 weeks. The best-corrected visual acuity was counting fingers in the right eye and 20/30 in the left eye. The fundus examination demonstrated a right retinal detachment inferiorly extending to the fovea and a left macular serous detachment. After multimodal imaging study, the patient was diagnosed as having a bullous variant of central serous chorioretinopathy and treated with oral spironolactone associated with adjuvant laser photocoagulation. The retinal changes resolved after 6 months. The final visual acuity was 20/20 in both eyes.

## Abstract (pt)

Relatamos o caso de um homem de 39 anos apresentando perda visual no olho direito há seis semanas. A melhor acuidade visual corrigida foi conta-dedos no olho direito e 20/30 no esquerdo. A fundoscopia demonstrou descolamento de retina direito inferiormente com extensão à fóvea e descolamento macular seroso à esquerda. Após estudos de imagem multimodal, o paciente foi diagnosticado com uma variante bolhosa de coriorretinopatia serosa central e tratado com espironolactona oral associada à fotocoagulação a laser adjuvante. As alterações retinianas resolveram após seis meses. A acuidade visual final foi 20/20 em ambos os olhos.

## INTRODUCTION

The bullous variant of central serous chorioretinopathy (bCSC) consists of a rare and severe form of chronic CSC first described by Gass in 1973^(1)^. The condition, which is most prevalent in men from their thirties to fifties, is characterized by exuberant fluid accumulation that leads to retinal detachments (RDs) of greater magnitude than those in the usual CSC^(2,3)^. In addition to bullous RDs, bCSC also manifests shifting of subretinal fluid during head position or scleral depression, multifocal pigment epithelial detachments, and exudative fibrinoid accumulation in the subretinal space^(1,3,4)^. Similar to acute and chronic CSC, bCSC has been related to psychological stress, subfoveal choroidal thickening (pachychoroid), and corticosteroid use^(2,5)^.

Treatment options for bCSC have been poorly explored in the literature and rely on lines applied to classic CSC, ranging from observation to systemic and topical medications. Administration of mineralocorticoid receptor antagonists such as eplerenone and spironolactone is aimed at containing fluid buildup and improving the patient’s vision. A single-case study previously described the use of spironolactone in bCSC with favorable results^(6)^. We report a second case of bCSC successfully treated with spironolactone and studied with multimodal imaging.

## CASE REPORT

A healthy 39-year-old man was referred for surgical treatment consideration after suspicion of rhegmatogenous retinal detachment in the right eye (OD). He complained of progressive blurry vision in both eyes, which was worse in the OD, for approximately 6 weeks. He had no history of trauma, medications, or medical conditions. In a systematic review, he reported recent episodes of emotional distress involving family issues.

**Figure 1.**
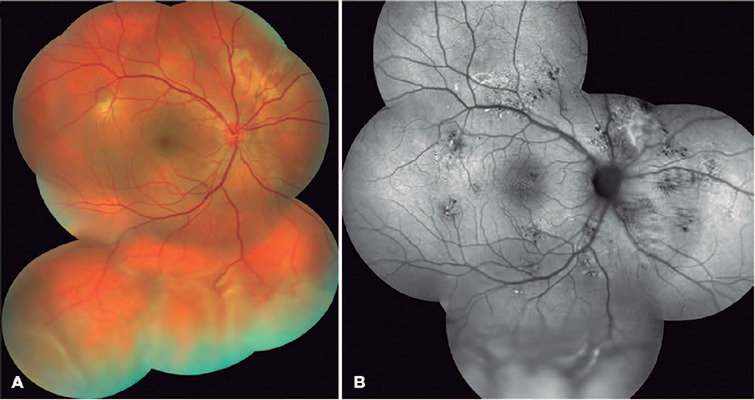
Color fundus picture mosaic of the right eye at presentation (A), showing subretinal exudation and fbrinoid deposits at the superior retina along with a bullous retinal detachment inferiorly. The fundus autofuorescence (B) shows points of hypoautofuorescence corresponding to the retinal pigment epithelium disturbance and hyperautofuorescence by the serous content accumulation.

**Figure 2.**
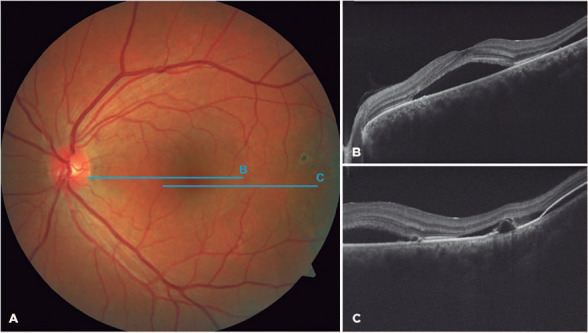
On the left eye, a retinal neurosensory detachment is notable at the central macula (A), which was confrmed by the spectral domain optical coherence tomography B-scan (B). The juxtafoveal scan (C) indicates various pigment epithelial detachments at the posterior pole.

On ocular examination, the best-corrected visual acuity (BCVA) was counting fingers in OD and 20/30 in the left eye (OS). The anterior segment was unremarkable. The intraocular pressures were within the normal range. The right fundus examination revealed a prominent bullous RD inferiorly extending to the macula and multiple areas of subretinal fibrinoid deposits in the posterior pole and mid-periphery, which were highlighted on the fundus autofluorescence (Figure 1). No peripheral breaks were detected under scleral indentation, although fluid shift indicative of a serous component was observed. The left fundus examination revealed serous macular detachment, multiple pigment epithelial detachments, and scattered pigmentary abnormalities (Figure 2). The patient’s vitals were assessed, revealing a normal systemic blood pressure.

**Figure 3.**
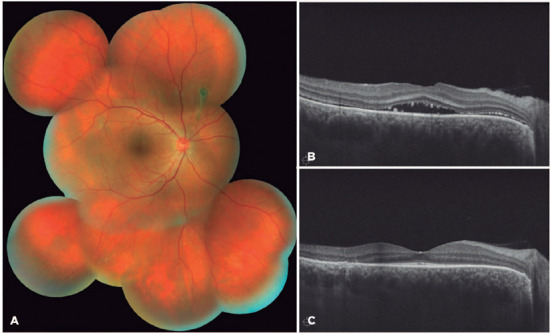
After 3 months of oral spironolactone, complete retinal reattachment was achieved, with cicatricial pigmentary changes in the right eye (A). The comparative optical coherence tomography B-scans through the right fovea demonstrate subretinal fuid improvement from baseline (B) to the third month after spironolactone (C). No adjuvant laser was delivered to the right eye.

**Figure 4.**
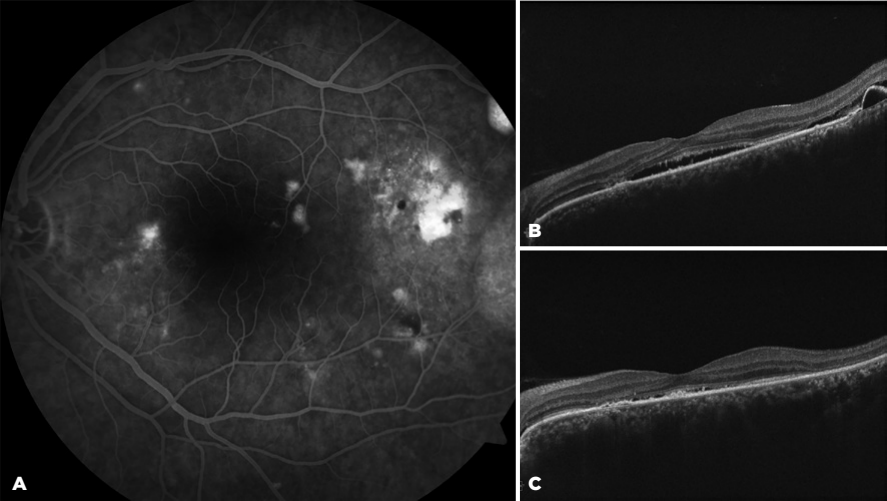
On the left eye, the leaking spot at the temporal macula persisted on fuorescein angiography (A) and optical coherence tomography (B) at the end of the third month of spironolactone. Focal laser photocoagulation was delivered at the site with fuid control at month 6 (C).

Accordingly, the patient was diagnosed as having bCSC and started receiving 50-mg oral spironolactone once a day for 1 month. At follow-up, he reported a major visual improvement but with residual visual distortion bilaterally. As some pockets of subretinal fluid were still noticeable on the fundus, the medication was continued for 2 more months with close surveillance. After the third month, complete retinal reattachment was achieved in the OD (Figure 3). A few active leaking spots were still detectable at the temporal macula of the OS on fluorescein angiography (Figure 4). The refractory area was thus treated with careful laser photocoagulation, and the spironolactone treatment was discontinued. On the sixth-month visit, no fluid relapse was present, and the final BCVA was 20/20 in both eyes. No adverse effect was reported.

## DISCUSSION

Bullous CSC is a rare manifestation of chronic CSC, causing exudative RDs secondary to massive fluid accumulation with visual compromise^(1,2,4)^. The bCSC variant is reported bilaterally in 15%-84%^(3)^ of cases, requiring differentiation from other causes of serous RD, such as Vogt-Koyanagi-Harada syndrome, multifocal choroiditis, malignant hypertension, posterior scleritis, choroidal metastasis, and uveal effusion syndrome^(5)^. In this case, the clinical presentation, imaging features, absence of intraocular inflammation, and thickened subfoveal choroid measurements of 453 µm in the OD and 512 µm in the OS reinforced the bCSC hypothesis.

Management of bCSC relies on the scarce information available in the literature, which mainly reports findings from small cohorts or single-case outcomes. Treatment strategies may be in accordance with those for classic CSC, including steroid discontinuation, antimineralocorticoid (aldosterone antagonist) drugs, laser photocoagulation, photodynamic therapy, and pars plana vitrectomy with internal drainage^(3,4,6,7)^. Generally, more than one approach is required to reach stability. Observation has been suggested in eyes with preserved BCVA^(4)^.

Oral spironolactone or eplerone has been used in CSC after studies have implicated a mineralocorticoid receptor mechanism in choroidal thickening and hyperpermeability^(5,7,8,9,10)^. Variable results have been reported as to its effectiveness in reducing fluid and preserving vision, particularly in the acute phase, which is considered by some authors as before 4 months. In a double-blind randomized placebo-controlled study, epleronone therapy did not prove to be superior to placebo in 57 treatment-naive patients with chronic CSC after 12 months of treatment^(10)^.

No investigation has particularly explored treatments for bCSC. In a single-case report, Ramos-Yao et al. described a favorable response in an individual with bCSC and symptoms that lasted for 1 week, the patient was treated with daily administration of 50-mg spironolactone for 4 months, followed by a maintenance dose of 25 mg/day^(6)^. In our case, the patient experienced visual deterioration for a longer period (6 weeks) and responded considerably after a single 3-month course of 50-mg/day spironolactone. Complimentary laser photocoagulation was useful at the final phase of treatment to control the late leaking spots outside the macula, allowing for medication discontinuation. While approximately 10% of patients treated with spironolactone may experience side effects, including headaches, diarrhea, fatigue, gynecomastia, or decreased libido^(7)^, no adverse issues were reported.

We recognize the limitations of this study concerning the conclusions and outcomes. The results obtained might have been due to other underlying factors, including spontaneous resolution despite medication. This may be addressed by future prospective investigations.
